# Topic “Application of Reproductive and Genomic Biotechnologies for Livestock Breeding and Selection”

**DOI:** 10.3390/ijms27083439

**Published:** 2026-04-11

**Authors:** Pedro M. Aponte, Manuel Garcia-Herreros

**Affiliations:** 1 Colegio de Ciencias Biológicas y Ambientales (COCIBA), Universidad San Francisco de Quito USFQ, Quito 170157, Ecuador; 2 Colegio de Ciencias de la Salud, Escuela de Medicina Veterinaria, Universidad San Francisco de Quito USFQ, Quito 170157, Ecuador; 3 Campus Cumbayá, Instituto de Investigaciones en Biomedicina “One-health”, Universidad San Francisco de Quito USFQ, Quito 170157, Ecuador; 4 Instituto Nacional de Investigação Agrária e Veterinária (INIAV), 2005-424 Santarém, Portugal; 5 CIISA-AL4AnimalS, Faculty of Veterinary Medicine, University of Lisbon (UL), 1300-477 Lisbon, Portugal

## 1. Introduction

The escalating global demand for animal products—meat, milk, and eggs—requires sustained advances in the efficiency, sustainability, and biological performance of livestock production systems. Traditionally, genetic improvement relied on phenotypic selection and pedigree analysis, methods that, although successful, are time-consuming and often limited by long generation intervals, the low heritability of traits, and the high costs of progeny testing [1]. The advent of molecular genetics/genomics and the subsequent reduction in genotyping costs have facilitated a paradigm shift in animal breeding, transitioning from conventional pedigree-based evaluations to molecular-based approaches [2]. 

Genomic selection (GS), first popularized by Meuwissen et al. [3], has revolutionized the breeding of cattle, swine, poultry, and aquaculture species. Unlike marker-assisted selection (MAS), which focuses on a limited number of quantitative trait loci (QTLs), GS uses dense, genome-wide markers—primarily single-nucleotide polymorphisms (SNPs)—to simultaneously evaluate thousands of markers, capturing both large and small genetic effects [4]. By facilitating the estimation of genomic estimated breeding values (GEBVs) in domestic animals, GS substantially reduces the generation interval and increases the intensity of selection, leading to rapid rates of genetic gain [5].

The success of GS is heavily dependent on the availability of a robust toolkit for managing large-scale genomic data, including high-density SNP arrays, whole-genome sequencing (WGS), and advanced bioinformatics, such as PLINK and SVS [6]. These tools enable precise quality control, genotype imputation, and pedigree verification. In parallel, statistical modeling has evolved from traditional linear models (e.g., GBLUP) to Bayesian approaches and emerging machine learning and deep learning techniques capable of handling complex, nonlinear relationships [7]. The integration of these tools enables the estimation of genetic merit for complex traits, including health, fertility, and production efficiency [8,9].

Despite the widespread adoption of GS, several challenges remain regarding the accuracy of prediction, especially in smaller populations; the integration of diverse "omics" data; and the lack of high-quality phenotypic data. This Research Topic provides comprehensive studies of the current genomic selection tools, gathering methodological innovation, functional genomics, and applied reproductive research across diverse livestock species.

## 2. Overview of Published Articles

### 2.1. Advances in Genomic Selection and Predictive Modeling

Contributions 2, 4, 6, 7, 13, 15, and 27 focus on increasing the accuracy, robustness, and practical applicability of genomic selection through methodological innovation and advanced statistical modeling. These studies collectively demonstrate that complex production, fertility, and conformation traits in livestock exhibit sufficient genetic variability to support effective genomic prediction strategies.

Several studies increased prediction accuracy by integrating prior information from genome-wide association analyses into genomic selection models. In contrast, other researchers explored weighted nonparametric and kernel-based regression methods designed to capture nonlinear genetic effects. The authors of contribution 27 further addressed optimizing marker density through low-density SNP panel design, emphasizing cost efficiency without substantial loss of predictive power. Contribution 15 illustrates how machine learning frameworks integrating genomic, phenotypic, and environmental data enhance prediction performance under field conditions. Together, these studies demonstrate the transition from traditional linear models toward integrative, data-driven genomic prediction systems capable of handling heterogeneous populations and complex traits.

Despite this methodological progress, a critical limitation shared among these contributions is their reliance on single-breed or genetically homogeneous reference populations, which constrains the generalizability of the proposed models. Prediction accuracies reported under controlled or breed-specific conditions may not hold when models are transferred to admixed, locally adapted, or numerically small populations, which represents a scenario common in many livestock systems outside major commercial breeds. Furthermore, although machine-learning- and kernel-based approaches show advantages in capturing nonlinear genetic effects, their interpretability and computational scalability under routine breeding program conditions remain insufficiently addressed. Future work should prioritize cross-population validation and the systematic benchmarking of emerging methods against established genomic prediction frameworks under realistic field conditions.

### 2.2. Functional Genomics of Reproductive and Developmental Traits

Contributions 3, 9, 11, 14, 16, 17, and 25 explore the genetic and molecular mechanisms underlying reproductive performance, gonadal function, and developmental regulation across multiple livestock species. These investigations moved beyond association toward functional interpretation, identifying regulatory variants and gene networks that influence fertility-related traits.

The authors of contribution 3 characterized the promoter activity affecting reproductive gene expression, whereas the authors of contributions 11 and 25 examined key signaling pathways and gene families involved in ovarian and reproductive tissue regulation. The transcriptomic profiling approaches outlined in contributions 14 and 17 identify differentially expressed genes and pathways associated with follicular development and tissue differentiation. In male reproductive biology, the researchers in contributions 9 and 16 elucidated the gene functions in Leydig cell function and spermatogenesis, highlighting the coordinated regulation of the endocrine, cell cycle, and metabolic pathways. Collectively, these studies represent a meaningful advance toward a mechanistic understanding of reproductive performance; however, most findings remain descriptive at the transcriptomic or gene expression level, lacking functional validation. Identifying differentially expressed genes or enriched pathways does not establish causality, and experimental evidence linking regulatory variants to phenotypic outcomes remains scarce. Additionally, the reliance on single time points, single tissues, or small sample sizes in several studies limits the robustness and reproducibility of the reported gene networks. Bridging this gap will require integrated approaches that combine functional genomics with gene editing or reporter assays as well as larger, multi-tissue, longitudinal datasets that capture the dynamic regulation of reproductive physiology across developmental stages.

### 2.3. Reproductive Biotechnologies and Gamete-Oriented Interventions

Contributions 5, 8, 20, and 22 address applied reproductive biotechnologies aimed at enhancing gamete quality, fertility preservation, and assisted reproduction. These studies emphasize the importance of optimizing biological conditions to safeguard genetic resources and improve reproductive efficiency.

The authors of contribution 8 evaluated antioxidant supplementation during semen processing, showing that targeted interventions can mitigate oxidative stress and increase post-thaw sperm functionality when applied at appropriate concentrations. The authors of contribution 22 extended the concept of genetic preservation to invertebrates by developing cryopreservation strategies for honey bee sperm, contributing to biodiversity conservation. The researchers in contribution 20 advanced in vitro induction systems for primordial germ cells, offering new tools for germline manipulation and functional studies. Contribution 5 highlights the interplay among phytochemicals, microbiota, and reproductive physiology, illustrating how nutritional and microbial modulation can influence fertility outcomes. Although these studies demonstrate the translational potential of gamete-oriented biotechnologies, a recurring limitation is the narrow experimental scope under which outcomes were evaluated. Studies on antioxidant supplementation and cryopreservation protocols, for instance, were often conducted under highly controlled laboratory conditions that do not reflect the variability inherent in field settings, hindering direct extrapolation to commercial reproductive management. Additionally, the absence of standardized outcome metrics among studies, such as consistent assessments of post-thaw sperm functionality or in vitro fertilization competence, limits cross-study comparability and the derivation of consensus recommendations, a problem further compounded by the scarcity of in vivo validation data confirming that laboratory-level increases in gamete quality translate into measurable gains in fertility outcomes under field conditions. Progress in this area will depend on larger, multicenter validation trials and the development of harmonized protocols that allow meaningful comparison across species, breeds, and reproductive management systems.

### 2.4. Gene Editing and High-Resolution Functional Screening

Contributions 10, 18, and 19 highlight the application of genome editing and high-throughput functional screening technologies in livestock research. The authors of contribution 10 identified genomic safe harbor loci suitable for stable transgene integration, demonstrating the feasibility of precise genomic modification without disrupting endogenous gene function. The researchers in contribution 19 applied CRISPR-based knockout screening to identify genes involved in hormone responsiveness, enabling systematic functional dissection of reproductive regulatory pathways.

Complementing these experimental studies, the authors of contribution 18 provides a comprehensive perspective on CRISPR-mediated genome editing in small ruminants, discussing its implications for genetic improvement and disease resistance. Gene editing technologies are valuable as functional genomics instruments within this Research Topic. However, their advancement toward routine application in commercial livestock breeding remains constrained by substantial regulatory and societal barriers. The regulatory landscape for CRISPR-based interventions considerably varies among jurisdictions, with the European Union maintaining strict GMO-equivalent classifications for most gene-edited organisms, whereas other regions have adopted more permissive frameworks, creating uncertainty for translational development and international trade of edited animals or germplasm. Beyond regulation, broader ethical questions regarding animal welfare, biodiversity risks, and equitable access to editing technologies have yet to be systematically addressed within livestock breeding frameworks. For gene editing to fulfill its potential beyond functional research, the field will need transparent regulatory harmonization and inclusive stakeholder engagement that extends beyond the scientific community.

### 2.5. Integrative Physiological, Population, and Systems-Level Perspectives

Contributions 1, 12, 21, 23, 24, and 26 expand the scope of this Research Topic beyond molecular analyses to encompass population genetics, enterprise-level modeling, and integrative physiological frameworks. The authors of contribution 1 investigated phylogeographic patterns and genetic diversity in wild populations, providing insights into the evolutionary background and genetic structure. The researchers in contributions 21 and 24 further examined genetic diversity and breed characterization, reinforcing the importance of conserving locally adapted genetic resources within modern breeding systems.

At the enterprise level, the authors of contribution 12 evaluated the economic implications of genetic improvement strategies, demonstrating how long-term genetic gain interacts with market dynamics and production management. The authors of contributions 23 and 26 explored the metabolic and adipogenic pathways associated with production traits, highlighting the intersection between genomic variation and physiological regulation. Collectively, these studies emphasize that the full impact of reproductive and genomic biotechnologies emerges when molecular innovation is integrated with population diversity, economic modeling, and production system considerations. The breadth of these contributions is a strength; however, these studies expose a persistent challenge: the integration of population-level diversity and economic modeling with molecular breeding tools remains more aspirational than operational. Studies on genetic diversity and breed characterization often operate in parallel with, rather than directly informing, genomic selection research, and the mechanisms through which locally adapted genetic resources can be preserved while being leveraged for genetic gain have not been resolved. The economic modeling presented in contribution 12, although valuable, also underscores the difficulty of predicting long-term genetic improvement under variable market and environmental conditions, a complexity that intensifies as climate change alters the environmental and resource conditions under which livestock production operates. Achieving this system-level vision will require coordinated data-sharing infrastructure, policy frameworks that incentivize conservation alongside productivity, and breeding models explicitly designed to balance short-term gain with long-term adaptive capacity.

## 3. Conclusions and Perspectives

### 3.1. Consolidated Insights from This Research Topic

The studies assembled in this Research Topic collectively demonstrate that reproductive and genomic biotechnologies have entered a phase of methodological maturity while achieving higher biological resolution and analytical complexity. Rather than functioning as isolated tools, genomic selection, functional genomics, reproductive biotechnologies, and advanced computational frameworks are converging into integrated breeding strategies, a trend that reflects both technical progress and a deeper recognition of the biological complexity underlying economically relevant traits.

Importantly, these findings reinforce a central working hypothesis of modern animal breeding: genetic merit cannot be fully understood or exploited without accounting for biological networks, environmental context, and management conditions. Fertility, growth, efficiency, and resilience emerge as polygenic, context-dependent traits shaped by interactions across multiple molecular and physiological layers. This Research Topic therefore highlights a shift away from reductionist models toward system-based interpretations of genotype–phenotype relationships.

### 3.2. Technological and Methodological Trajectories in Genomic Selection

Technological progress in genomic selection is increasingly characterized by refinement rather than replacement. The main impact of high-density genotyping and whole-genome sequencing lies not in marker resolution *per se* but in enabling biologically informed modeling strategies beyond purely statistical associations [10]. Single-step methodologies exemplify this transition by increasing prediction accuracy while maintaining compatibility with established breeding infrastructure [4].

The expansion toward across-breed genomic selection and two-dimensional selection frameworks reflects a broader recognition that robustness across populations, environments, and production goals cannot be achieved through within-breed optimization alone [11,12,13]. Collectively, these methodological trajectories reflect a shift from static evaluation models toward flexible, population-aware breeding strategies that account for genetic diversity and environmental heterogeneity.

Whether these approaches will deliver consistent gains across the full spectrum of livestock diversity, including numerically small and locally adapted breeds, remains an open and practically important question. Collectively, these trajectories point toward flexible, population-aware breeding strategies, although their real-world performance will ultimately depend on the quality and diversity of the reference populations underpinning them.

### 3.3. Expansion Through Emerging -Omics and Functional Genomics

The growing role of functional genomics and emerging -omics technologies represents one of the most consequential and analytically demanding developments documented in this Research Topic, shifting genomic selection from correlation-based prediction toward mechanism-informed decision-making [14,15]. Transcriptomics, proteomics, and metabolomics provide complementary layers of information that reveal how genetic variation translates into cellular function, physiological regulation, and ultimately phenotypic expression [16].

Epigenomic regulation, particularly DNA methylation, represents an additional dimension that helps explain phenotypic plasticity, environmental adaptation, and intergenerational effects that are not fully captured by DNA sequence variation alone [17,18]. Microbiome research further expands the concept of the effective genome to include host-associated microbial communities with demonstrable influence on nutrition, immunity, and reproductive performance [19,20]. Taken together, these -omics layers redefine breeding targets as dynamic biological systems rather than fixed genetic attributes, a reconceptualization with wide implications for trait definition, model design, and the biological interpretation of genomic predictions. The challenge ahead lies in integrating these data streams using methods that are analytically tractable, reproducible across populations, and actionable within breeding programs.

### 3.4. Genome Editing as a Tool for Functional Validation

Genome editing technologies, particularly CRISPR-based systems, occupy a pivotal role in the transition from discovery to validation [21]. Within this Research Topic, gene editing primarily emerges as a tool for the direct testing of gene function, regulatory mechanisms, and causal pathways underlying complex traits. This role is already large and will likely expand as genomic association studies achieve finer resolution and the demand for causal rather than correlational interpretation of genomic findings intensifies. 

The value of genome editing for functional annotation, reproductive biology research, and model development is already substantial [22]; the translation of genome editing into commercial breeding, however, remains contingent on regulatory harmonization and broader societal acceptance, issues addressed in detail in Section 3.7. By enabling the targeted manipulation of candidate genes and regulatory elements, genome editing strengthens the evidentiary foundation of genomic selection and supports more informed prioritization of breeding targets, a contribution that benefits the field regardless of the pace of regulatory progress.

### 3.5. Computational Intelligence and System-Level Integration

As data generation accelerates, computational capacity and analytical sophistication have become central determinants of progress in livestock genomics. Artificial intelligence, machine learning, and deep learning models offer tools for capturing nonlinear relationships and extracting actionable patterns from high-dimensional datasets [23]. Convolutional neural networks and automated phenotyping pipelines are beginning to expand the scope and throughput of phenotypic data collection, potentially alleviating one of the field’s most persistent bottlenecks: the scarcity of large-scale, high-quality phenotypic data [24].

Emerging frameworks such as IoT-enabled sensors, digital twins, and genomic-enviromics approaches point toward real-time, adaptive breeding systems that integrate genomic information with continuous environmental and management data [25,26]. These developments represent a meaningful shift from retrospective evaluation toward dynamic decision support, although their operational deployment at scale raises unresolved questions about data standardization, infrastructure requirements, and interoperability across breeding systems. Looking further ahead, quantum computing concepts highlight the growing need for novel computational solutions as biological datasets continue to increase in volume and complexity [27], a frontier that warrants early investment in algorithm development and computational infrastructure, even if practical breeding applications remain distant.

### 3.6. Toward Integrated, Adaptive Breeding Systems

The evidence collective from this Research Topic supports a future vision in which livestock breeding operates as an integrated, adaptive system, one where genomic selection is continuously informed by multiomics data, functional validation, advanced computation, and real-time phenotyping. Rather than maximizing single traits, next-generation breeding strategies will need to explicitly balance resilience, reproductive efficiency, sustainability, and long-term genetic diversity, goals that are not always aligned and that will require deliberate trade-off management within breeding program design.

Ultimately, the convergence of reproductive and genomic biotechnologies represents not merely technological advancement but a conceptual transformation in how genetic improvement is pursued and justified. Realizing this transformation will depend on continued technical innovation and remarkably on the governance structures, data-sharing frameworks, and institutional commitments needed to make integrated breeding systems equitable, reproducible, and globally applicable.

### 3.7. Technical Limitations, Implementation Barriers, and Regulatory Constraints

Despite the methodological advances documented in this Research Topic, genomic selection and associated biotechnologies remain subject to a set of interconnected technical and practical limitations that constrain their broader applicability. Perhaps the most persistent bottleneck is the scarcity of high-quality phenotypic data: although genotyping has become fast and affordable, obtaining high-quality, standardized phenotypic data at scale remains costly and logistically demanding. This challenge is especially acute for traits with low observability, such as feed efficiency, disease resilience, or fertility under field conditions. This asymmetry between genotypic and phenotypic data availability directly compromises model accuracy and limits the biological relevance of genomic predictions.

Genotype-by-environment interactions represent a further complication, as trait expression varies across production environments in ways that reduce the reliability of predictions when models trained in one context are applied to another. Closely related is the challenge of model transferability across populations: differences in genetic structure, allele frequencies, and linkage disequilibrium patterns mean that genomic prediction equations developed in large, well-resourced reference populations often perform poorly when extended to numerically smaller, locally adapted, or genetically distinct breeds. Addressing these limitations requires not only larger and more diverse reference populations but also deliberate model design that accounts for population-specific genomic architecture.

At the implementation level, the practical deployment of genomic selection demands sustained infrastructure, such as long-term phenotypic recording systems, computational capacity, and institutional continuity, which remains unevenly distributed across production systems and world regions, raising concerns about equitable access to genomic tools and the risk of widening existing technological gaps between well-resourced and resource-limited livestock sectors.

Regulatory and ethical considerations add a further layer of complexity, particularly where genomic selection intersects with gene editing technologies. The regulatory landscape for CRISPR-based interventions remains fragmented across jurisdictions: the European Union classifies most gene-edited organisms under stringent GMO-equivalent frameworks, whereas countries such as the United States, Brazil, and Argentina have adopted more permissive, product-based regulatory approaches. This jurisdictional heterogeneity creates uncertainty for translational development, complicates the international trade of edited animals and germplasm, and may discourage investment in species or production systems that operate across multiple regulatory environments. Beyond regulation, broader societal questions, which include data ownership, animal welfare implications of accelerated genetic change, and the concentration of genomic resources among a small number of commercial entities, warrant transparent, inclusive dialogue that extends well beyond the scientific community. The responsible advancement of these technologies will ultimately depend as much on governance frameworks and stakeholder engagement as on continued technical innovation.

## Data Availability

Data sharing is not applicable to this article.
